# Assessing standardized contrast effects in ANCOVA: Confidence intervals, precision evaluations, and sample size requirements

**DOI:** 10.1371/journal.pone.0282161

**Published:** 2023-02-24

**Authors:** Gwowen Shieh

**Affiliations:** Department of Management Science, National Yang Ming Chiao Tung University, Hsinchu, Taiwan; Universita degli Studi di Milano, ITALY

## Abstract

Standardized effect sizes and confidence intervals are useful statistical assessments for comparing results across different studies when measurement units are not directly comparable. This paper aims to describe and compare confidence interval estimation methods for the standardized contrasts of treatment effects in ANCOVA designs. Sample size procedures are also presented to assure that the resulting confidence intervals yield informative estimation with adequate precision. Exact interval estimation approach has theoretical and empirical advantages in coverage probability and interval width over the approximate interval procedures. Numerical investigations of the existing method reveal that the omission of covariate variables has a negative impact on sample size calculations for precise interval estimation, especially when there is disparity in influential covariate variables. The proposed approaches and developed computer programs fully utilize covariate properties in interval estimation and provide accurate sample size determinations under the precision considerations of the expected interval width and the assurance probability of interval width.

## Introduction

The utility of effect sizes and confidence intervals has been strongly emphasized in several editorial guidelines and methodological implications. The standardized mean difference between two independent populations is the most frequently used effect size measure across virtually all disciplines of scientific researches. Accordingly, the intuitive formula Hedges’s [1] *g* or commonly known as of Cohen’s [2] *d* is an estimate of the population standardized mean difference and is defined as the difference between two sample means divided by their pooled sample standard deviation under homoscedasticity. Unlike the unstandardized contrasts, the effect size reporting and interpretation practices suggest that the standardized effect sizes are useful when comparing results from multiple studies using measurement instruments whose raw units are not directly comparable. Comprehensive expositions and practical uses about standardized mean difference and related effect size measures are available in Grissom and Kim [3], Kline [4], Lin and Aloe [5], Takeshima et al. [6], Zhang [7], Zhang and Heyse [8], and the references therein.

Considerable attention has also been drawn toward the development of interval estimation methods for standardized mean difference, see Odgaard and Fowler [9], Smithson [10], Steiger and Fouladi [11], Tian [12], Wu, Jiang and Wei [13], and Zou [14], among others. Standardized contrasts of treatment effects and interval procedures are presented in Kline [4], Steiger and Fouladi [11], and Steiger [15] in the context of ANOVA. Moreover, Grissom and Kim [3], Kline [4], Levin [16], and Olejnik and Algina [17] addressed the calculation and interpretation of standardized contrasts of treatment effects in ANCOVA. However, these studies did not discuss the issues of interval estimation of standardized treatment contrasts. The exceptional case of Lai and Kelley [18] presented confidence interval formula and sample size determination of standardized contrast effects for ANCOVA designs with only one covariate. Particularly, Lai and Kelley [18] focused on the special cases of ANCOVA with a single covariate and suggested that, under randomized designs, the linear contrast of covariate means is usually close to zero. Hence, the corresponding covariate quantity can be omitted in the sample variance of a linear contrast for confidence interval and sample size calculations of standardized treatment contrasts. Despite the conceptual argument and technical simplification, they did not conduct empirical study to justify the suggested procedures regarding the influence of omitted covariate variable. Hence, it is of methodological importance to perform detailed numerical examination for clarifying the adequacy of the approximate confidence interval and sample size methods. Moreover, their model setting did not cover the more involved situations with more than one covariate variable. Thus, it is of practical importance to present more accurate interval estimation and sample size procedures for ANCOVA studies with possibly diverse covariate configurations.

Although randomized experiments are preferred in the gold standard of research designs, it is still possible that randomized experiments may generates imbalanced groups in terms of sample size and baseline disparities even in large studies. The related results about the impact of covariate imbalance on power of ANCOVA studies and randomized clinical trials can be found in Ciolino et al. [19,20], Egbewale, Lewis and Sim [21], Kahan and Morris [22] and the references therein. More importantly, feasible power and sample size procedures were developed in Shieh [23–25] and Tang [26–28] to accommodate covariate randomness and imbalance for randomized and nonrandomized designs. It should be emphasized that a thorough and rigorous illustration of interval estimation, precision assessment, and sample size techniques for standardized contrast effects of ANCOVA has not been given in the literature.

In view of the limited findings in the literature, the goal of the current article is twofold. First, this research examines and compares exact and approximate confidence interval procedures of standardized contrast effects for ANCOVA designs with one or more covariate variables. A second aim of the present study is to extend and complement the demonstration of unstandardized contrast effects in Shieh [29] by developing exact precision and sample size procedures for standardized contrast effects under the assumption of multinormal covariate variables. Extensive numerical studies for interval estimation, precision assessment, sample size determination were conducted to illustrate the advantages of the proposed techniques over the approximate methods. In order to facilitate data analysis and design planning, computer algorithms are presented to resolve the computational issues of the suggested procedures.

## Methods

### Interval estimation procedures

Consider the fixed-effects ANCOVA model with *G* treatment groups and *P* multiple covariates:

$$Y_{ij}=\mu_{i}+\Sigma_{k=1}^{P}X_{kij}\beta_{k}+\epsilon_{ij},$$ (1)

where *Y*_*ij*_ is the score of the *j*th subject in the *i*th treatment group on the response variable, μ_*i*_ is the *i*th intercept, *X*_*kij*_ is the score of the *j*th subject in the *i*th treatment group on the *k*th covariate, β_*k*_ is the slope coefficient of the *k*th covariate, and ϵ_*ij*_ is the independent *N*(0, *σ*^2^) error with *i* = 1,…, *G* (≥ 2), *j* = 1, …, *N*_*i*_, and *k* = 1, …, *P* (≥ 1).

In view of the importance and utility advocated in Grissom and Kim [4], Kline [5], and Levin [18], the current study focuses on the appraisals of standardized contrasts rather than the conventional or unstandardized contrasts. A standardized linear contrast of adjusted group means $\left\{\mu_{1}^{*},\ldots ,\mu_{G}^{*}\right\}$ is defined as

$$\psi^{*}=\;\frac{\psi }{\;\sigma },$$ (2)

where $\psi =\sum_{i=1}^{G}c_{i}\mu_{i}^{*}$, *c*_*i*_ is the linear coefficient with $\sum_{i=1}^{G}c_{i}=0,\;\mu_{i}^{*}=\mu_{i}+\sum_{k=1}^{P}{\overset{-}{X}}_{k\cdot \cdot }\beta_{k}$ is the expected group response evaluated at the grand covariate means ${\overset{-}{X}}_{k\cdot \cdot }=\sum_{i=1}^{G}\sum_{j=1}^{N_{i}}X_{kij}/N_{T}$, and $N_{T}=\sum_{i=1}^{G}N_{i}$. Note that a linear contrast of adjusted group means is equivalent to a linear contrast of intercept parameters $\psi =\sum_{i=1}^{G}c_{i}\mu_{i}^{*}=\sum_{i=1}^{G}c_{i}\mu_{i}$ because $\sum_{i=1}^{G}c_{i}=0$.

The standard results in Rencher and Schaalje [30] show that the least square estimator for the *i*th intercept μ_*i*_ is given by

$${\hat{\mu }}_{i}={\bar{Y}}_{i}-\sum_{i=1}^{P}{\bar{X}}_{ki\cdot }{\hat{\beta }}_{k},$$ (3)

where ${\overset{-}{Y}}_{i}=\sum_{j=1}^{N_{i}}Y_{ij}/N_{i},\;\hat{\beta }={\left({\hat{\beta }}_{1},\ldots ,\;{\hat{\beta }}_{P}\right)}^{\text{T}}=S_{XX}^{-1}S_{XY},\;S_{XX}=\sum_{i=1}^{G}\sum_{j=1}^{N_{i}}{\left(X_{ij}-{\overset{-}{X}}_{i})(X_{ij}-{\overset{-}{X}}_{i}\right)}^{\text{T}}$, $S_{XY}=\sum_{i=1}^{G}\sum_{j=1}^{N_{i}}{\left(X_{ij}-{\overset{-}{X}}_{i}\right)\left(Y_{ij}-{\overset{-}{Y}}_{i}\right)}^{\text{T}}$, $X_{ij}={\left(X_{1ij},\ldots ,X_{Pij}\right)}^{\text{T}}$, ${\overset{-}{X}}_{i}=\sum_{j=1}^{N_{i}}X_{ij}/N_{i}={\left({\overset{-}{X}}_{1i\cdot },\ldots ,{\overset{-}{X}}_{Pi\cdot }\right)}^{\text{T}}$, and ${\overset{-}{X}}_{ki\cdot }=\sum_{j=1}^{N_{i}}X_{kij}/N_{i}$. Moreover, an unbiased estimator of the adjusted group mean $\mu_{i}^{*}$ is of the form

$${\hat{\mu }}_{i}^{*}={\hat{\mu }}_{i}+\Sigma_{k=1}^{P}{\bar{X}}_{k\cdot \cdot }{\hat{\beta }}_{k}={\bar{Y}}_{i}-\Sigma_{k=1}^{P}{\hat{\beta }}_{k}\left({\bar{X}}_{ki\cdot }-{\bar{X}}_{k\cdot \cdot }\right).$$ (4)

Thus, a convenient estimator $\hat{\psi }$ for the contrast effect ψ is

$$\hat{\psi }=\Sigma_{i=1}^{G}c_{i}{\hat{\mu }}_{i}^{*}=\Sigma_{i=1}^{G}c_{i}{\hat{\mu }}_{i}.$$ (5)

It can be shown that the linear contrast estimator has the distribution

$$\hat{\psi }\;\sim N\left(\psi ,\tau^{2}\right),$$ (6)

where $\tau^{2}=Var\left(\hat{\psi }\right)=\sigma^{2}V$, *V* = *a* + *Q*/(*N*_*T*_−*G*), $a=\sum_{i=1}^{G}c_{i}^{2}/N_{i},\;{Q=(\sum_{i=1}^{G}c_{i}{\overset{-}{X}}_{i})}^{\text{T}}V_{XX}^{-1}(\sum_{i=1}^{G}c_{i}{\overset{-}{X}}_{i})$, and ***V***_*XX*_ = ***S***_*XX*_/(*N*_*T*_ − *G*). Note that the magnitude *Q* represents an estimate of the degree of covariate disparity in terms of the linear contrast of standardized covariate means.

The confidence interval of the contrast effect ψ can be obtained by the standard transformation *T* and associated distribution:

$$T=\frac{\hat{\psi }–\psi }{\hat{\tau }}\sim t(\nu ),$$ (7)

where ${\hat{\tau }}^{2}={\hat{\sigma }}^{2}V,\;{\hat{\sigma }}^{2}=SSE/\nu ,\;SSE=\sum_{i=1}^{G}\sum_{j=1}^{N_{i}}{\left(Y_{ij}-{\overset{-}{Y}}_{i}\right)}^{2}-S_{XY}^{\text{T}}S_{XX}^{-1}S_{XY}$, and ν = *N*_*T*_ − *G* − *P*, and *t*(ν) is the *t* distribution with degrees of freedom ν. Specifically, the 100(1 –α)% equal-tail two-sided confidence limits (${\hat{\psi }}_{L},\;{\hat{\psi }}_{U}$) of the contrast effect ψ are

$${\hat{\psi }}_{L}=\hat{\psi }–t_{\nu ,\alpha /2}\hat{\tau }\;\text{and}\;{\hat{\psi }}_{U}=\hat{\psi }\;+t_{\nu ,\alpha /2}\hat{\tau },$$ (8)

where *t*_ν,α/2_ is the upper 100(α/2) percentile of the *t*(ν) distribution.

### Confidence intervals

A natural estimator of the standardized contrast ψ* is

$${\hat{\psi }}^{*}=\;\frac{\hat{\psi }}{\;\hat{\sigma }}.$$ (9)

Note that the well-known standardized mean difference index Hedges’s [1] *g* or Cohen’s [2] *d* provides a slight overestimate of the standardized mean difference δ = (*μ*_*E*_ − *μ*_*C*_)/*σ* between the experimental and control groups. Similarly, the standardized contrast formula ${\hat{\psi }}^{*}$ is a positively biased estimator of ψ*. Following the model assumption, $K={\nu \hat{\sigma }}^{2}/\sigma^{2}\;\sim \;\chi^{2}(\nu )$, where χ^2^(*ν*) is a chi-square distribution with degrees of freedom *ν*. Standard derivations show that *E*[*K*^–1/2^] = {2^−1/2^Γ[(ν − 1)/2]}/Γ(ν/2) and $E=[1/\hat{\sigma }]=(1/\sigma )\{{\left(\nu /2\right)}^{1/2}\Gamma [(\nu -1)/2]\}/\Gamma (\nu /2)$ for *ν* > 1 and Γ{·} is the gamma function. Because $\hat{\psi }$ and ${\hat{\sigma }}^{2}$ are independent, an unbiased estimator of ψ* can be obtained as ${\hat{\psi }}_{UB}^{*}=u{\hat{\psi }}^{*}$ where *u* = Γ(ν/2)/{(ν/2)^1/2^Γ[(ν − 1)/2]} < 1.

Moreover, the standardized statistic T* has the distribution

$$T^{*}=\frac{\hat{\psi }}{\;\hat{\tau }}\sim t\left(\nu ,\;\lambda \right),$$ (10)

where *t*(ν, λ) is a noncentral *t* distribution with degrees of freedom ν and noncentrality parameter λ = ψ*/*V*^1/2^. The fundamental properties and related applications of noncentral *t* distribution are available in Johnson, Kotz, and Balakrishnan [31, Chapter 31]. Then, the noncentrality inversion procedure of Casella and Berger [32, Section 9.2.3], Mood, Graybill and Boes [33, Section 4.2], and Venables [34] provide the exact confidence interval estimations of ψ*.

Accordingly, the upper 100(1 –*α*_1_)% confidence interval of ψ* is of the form (${\hat{\psi }}_{EL}^{*}$, ∞), in which ${\hat{\psi }}_{EL}^{*}$ satisfies

$$P\{t(\nu ,\;{\hat{\psi }}_{EL}^{*}/V^{1/2})<T_{O}^{*}\}=1-\alpha_{1},$$ (11)

where $T_{O}^{*}$ is the observed value of T*. On the other hand, the lower 100(1 –*α*_2_)% confidence interval of ψ* is of the form (–∞, ${\hat{\psi }}_{EU}^{*}$) in which ${\hat{\psi }}_{EU}^{*}$ satisfies

$$P\{t(\nu ,\;{\hat{\psi }}_{EU}^{*}/V^{1/2})>T_{O}^{*}\}=1-\alpha_{2}.$$ (12)

Furthermore, a 100(1 –*α*)% two-sided confidence interval {${\hat{\psi }}_{EL}^{*},{\hat{\psi }}_{EU}^{*}$} of ψ* can be obtained by jointly applying Eqs 11 and 12 with *α*_1_ = *α*_2_ = α/2 so that

$$P\{{\hat{\psi }}_{EL}^{*}<\psi^{*}<{\hat{\psi }}_{EU}^{*}\}=1-\alpha .$$ (13)

The particular method has been demonstrated for obtaining exact confidence intervals of standardized mean difference effect size in Kline [4], Odgarrd and Fowler [9], Smithson [10], and Steiger and Fouladi [11]. Notably, the confidence interval {${\hat{\psi }}_{EL}^{*},{\hat{\psi }}_{EU}^{*}$} does not have a convenient closed-form expression. The numerical computations of exact confidence intervals require the evaluation of the cumulative distribution function of a noncentral *t* variable. The corresponding SAS/IML and R computer programs for performing the exact confidence interval calculations are available as supplementary material.

It is shown in Johnson, Kotz, and Balakrishnan [31, p. 513] for large degrees of freedom that *E*[*t*(ν, λ)] ≐ λ and *Var*[*t*(ν, λ)] ≐ 1 + λ^2^/(2ν). The results suggest that $T^{*}\dot{\sim }N(\lambda ,\;1+\lambda^{2}/(2\nu ))$ for large samples. Hence, the standardized contrast estimator ${\hat{\psi }}^{*}=V^{1/2}T^{*}$ has a asymptotic distribution

$${\hat{\psi }}^{*}\dot{\sim }N(\psi^{*},\;\tau^{*2}),$$ (14)

where τ*^2^ = *V* + ψ*^2^/(2ν). Using the asymptotic property, the approximate 100(1 –*α*)% equal-tail two-sided confidence limits {${\hat{\psi }}_{AL}^{*},{\hat{\psi }}_{AU}^{*}$} of ψ* can be obtained as

$${\hat{\psi }}_{AL}^{*}={\hat{\psi }}^{*}–z_{\alpha /2}{\hat{\tau }}^{*}\;\text{and}\;{\hat{\psi }}_{AU}^{*}={\hat{\psi }}^{*}+z_{\alpha /2}{\hat{\tau }}^{*},$$ (15)

where ${\hat{\tau }}^{*2}=V+{\hat{\psi }}^{*2}/(2\nu )$ and *z*_α/2_ is the upper 100(*α*/2) percentile of the standard normal distribution. The same technique was previously recommended in Hedges [1] to construct confidence intervals for the population standardized mean difference δ.

The functional relationship between the unstandardized and standardized contrast effects ψ and ψ* = ψ/σ suggests approximate confidence interval of ψ* can be immediately obtained by dividing the confidence limits {${\hat{\psi }}_{L},{\hat{\psi }}_{U}$} of ψ by the standard error estimate $\hat{\sigma }$. This direct division gives a approximate 100(1 –*α*)% equal-tail two-sided confidence interval {${\hat{\psi }}_{DL}^{*},{\hat{\psi }}_{DU}^{*}$} of ψ* where

$${\hat{\psi }}_{DL}^{*}={\hat{\psi }}^{*}–t_{\nu ,\alpha /2}V^{1/2}\;\text{and}\;{\hat{\psi }}_{DU}^{*}={\hat{\psi }}^{*}+t_{\nu ,\alpha /2}V^{1/2}.$$ (16)

The identical procedure was proposed in Bird [35] and Fidler and Thompson [36] for finding the confidence interval of δ from the confidence limits of mean difference *μ*_*E*_ − *μ*_*C*_, and in Kline [4] for obtaining confidence interval of standardized treatment contrast from the interval endpoints of treatment contrast in ANOVA.

It should be noted that the exact two-sided interval estimates {${\hat{\psi }}_{EL}^{*},{\hat{\psi }}_{EU}^{*}$} are not equidistant from the standardized contrast estimate ${\hat{\psi }}^{*}$ except for the special case ${\hat{\psi }}^{*}=0$. However, the two approximate confidence intervals {${\hat{\psi }}_{AL}^{*},{\hat{\psi }}_{AU}^{*}$} and {${\hat{\psi }}_{DL}^{*},{\hat{\psi }}_{DU}^{*}$} are equidistant around the estimate ${\hat{\psi }}^{*}$. The two intervals {${\hat{\psi }}_{AL}^{*},{\hat{\psi }}_{AU}^{*}$} and {${\hat{\psi }}_{AL}^{*},{\hat{\psi }}_{AU}^{*}$} have the half-width $H_{A}=z_{\alpha /2}{\left\{V+{\hat{\psi }}^{*2}/(2\nu )\right\}}^{1/2}$ and *H*_*D*_ = *t*_ν,α/2_*V*^1/2^, respectively. Note that the difference between *z*_α/2_ and *t*_ν,α/2_ is usually trivial for large ν. Hence, it is generally true that *H*_*D*_ ≤ *H*_*A*_ and the interval {${\hat{\psi }}_{DL}^{*},{\hat{\psi }}_{DU}^{*}$} is practically within the interval {${\hat{\psi }}_{AL}^{*},{\hat{\psi }}_{AU}^{*}$} even when the estimate ${\hat{\psi }}^{*}$ is nearly zero. To contribute to the understanding of interval estimation of the standardized contrast effects, numerical appraisals are presented next to investigate the estimation behavior of the three different confidence intervals.

### An example

Fleiss [37, Section 7.2] described a study comparing three methods of treating the learning disabilities of children with respiratory diseases. The response variable is the number of correct answers to a test with 15 questions, and the covariate variable is the number of correct answers to a similar test with 7 questions. It is noted that the proportions of correct answers were not close to zero or one. Hence, no further transformation was applied to transform the discrete data for satisfying the normal assumption. The adjusted mean estimates and group sample sizes are $\{{\hat{\mu }}_{1}^{*},\;{\hat{\mu }}_{2}^{*},\;{\hat{\mu }}_{3}^{*}\}=\left\{5.7408,\;8.5512,\;7.8950\right\}$ and {*N*_1_, *N*_2_, *N*_3_} = {19, 20, 20}, respectively. Also, the covariate coefficient estimate is $\hat{\beta }=1.2946$ and the sample variance is ${\hat{\sigma }}^{2}=3.2728$. The omnibus *F* test of no treatment effect is F* = 12.52 with a *p*-value < 0.0001. Accordingly, the null hypothesis of treatment effects is rejected and there exists statistically significant differences among the three treatments at α = 0.05.

For illustration, standardized contrast analysis is presented here to complement the conventional unstandardized contrast assessments. With {*c*_1_, *c*_2_, *c*_3_} = {–1, 1/2, 1/2}, the unstandardized and standardized contrast estimates are $\hat{\psi }=2.4853$ and ${\hat{\psi }}^{*}=1.3721$, respectively. It can be shown that the linear contrast of standardized covariate means or the covariate disparity index is *Q* = 0.2131 and the factor *V* of the variance of the linear contrast is *V* = 0.0814. The 90% confidence intervals of the exact approach, asymptotic method, and direct division are {0.8504, 1.8827}, {0.8558, 1.8885}, and {0.8947, 1.8496}, respectively. Moreover, the corresponding 95% confidence intervals of the three procedures are {0.7519, 1.9820}, {0.7568, 1.9874}, and {0.8002, 1.9440}. These results suggest that the exact approach and asymptotic method give very similar confidence limits and they evidently differ from the narrowest interval constructed by direct division.

The computations of the exact confidence intervals can be performed with the supplemental SAS/IML and R programs. Researchers only need to provide the desired key settings to replace the prescribed exemplifying values in the computer codes. Specifically, the programming lines of the SAS supplemental file A for computing confidence interval of standardized contrast are as follows:

PROC IML;
*USER SPECIFICATION PORTION;
*DESIGNATED ALPHA;ALPHA = 0.10;
*NUMBER OF GROUPS;G = 3;
*NUMBER OF COVARIATES;P = 1;
*TOAL SAMPLE SIZE;NT = 59;
*CONTRAST ESTIMATE;PSIH = 2.4823;
*SAMPLE VARIANCE;SIGSQH = 3.2728;
*FACTOR FOR THE VARIANCE OF A LINEAR CONTRAST TAUSQ;V = 0.081437;
*END OF USER SPECIFICATION PORTION;

The R supplemental program D has similar specifications:

ancova.scie.apx1.fun(alpha = 0.1, g = 3, p = 1, nt = 59, psih = 2.4823, sigsqh = 3.2728, v = 0.081437).

### Empirical comparisons

To provide a more thorough evaluation, simulation studies were performed to examine the coverage performance of the interval procedures. On the other hand, the more involved precision assessments and sample size determinations of standardized contrast effects are presented in the Results section. For ease of explication, the model and parameter configurations of the learning disabilities study are modified and extended in this numerical examination. Specifically, Monte Carlo simulation studies of 10,000 iterations were performed with the normal distribution of the linear contrast $\hat{\psi }\sim N(\psi ,\tau^{2})$ and the chi-square distribution of the model variance $K={\nu \hat{\sigma }}^{2}/\sigma^{2}\;\sim \;\chi^{2}(\nu )$. Note that the variance of a linear contrast has the simple form τ^2^ = σ^2^*V* and $V=\Sigma_{i=1}^{3}c_{i}/N_{i}+Q/(N_{T}–3)$. Accordingly, the error variance estimate and covariate statistic of the learning disabilities data are set as the variance component σ^2^ = 3.2728 and covariate disparity *Q* = 0.2131. The contrast effect ψ for the contrast coefficients {*c*_1_, *c*_2_, *c*_3_} = {–1, 1/2, 1/2} has four different magnitudes so that ψ = ψ* = ψ/σ = 0, 1, 2, and 3. Also, the designated balanced designs with *G* = 3 have the group sample size *N* = 5, 10, and 20. For each replicate, the lower and upper confidence limits {${\hat{\psi }}_{EL}^{*},\;{\hat{\psi }}_{EU}^{*}\},\;\{{\hat{\psi }}_{AL}^{*},\;{\hat{\psi }}_{AU}^{*}$} and {${\hat{\psi }}_{DL}^{*},\;{\hat{\psi }}_{DU}^{*}$} were computed for the 95% and 97.5% one-sided confidence intervals. These interval limits are then combined to construct the 90% and 95% equal-tail two-sided confidence intervals. The simulated coverage probability was the proportion of the 10,000 replicates whose confidence interval contained the population standardized contrast effect. The adequacy of the one- and two-sided interval procedures is determined by the error between the simulated coverage probability and the nominal coverage probability. The results of the 12 combinations of total sample size and standardized contrast effect are summarized in Tables 1 and 2 for the two-sided confidence coefficient 1 –α = 0.90 and 0.95, respectively.

**Table 1:** The error between simulated coverage probability and nominal coverage probability of the 90% two-sided and 95% one-sided confidence intervals for the number of groups *G* = 3, one covariate *P* = 1, contrast coefficients {–1, 1/2, 1/2}, error variance σ^2^ = 3.2728, and balanced designs with *N*_*T*_ = 15, 30, and 60.

		Exact approach			Asymptotic method			Direct division		
*N*_*T*_	ψ*	Upper 95% CI	Lower 95% CI	Two-sided 90% CI	Upper 95% CI	Lower 95% CI	Two-sided 90% CI	Upper 95% CI	Lower 95% CI	Two-sided 90% CI
15	0	–0.0008	–0.0019	–0.0027	–0.0045	–0.0044	–0.0089	–0.0008	–0.0019	–0.0027
	1	0.0037	–0.0011	0.0026	–0.0001	–0.0006	–0.0007	–0.0297	0.0129	–0.0168
	2	0.0004	0.0031	0.0035	–0.0046	0.0062	0.0016	–0.0815	0.0087	–0.0728
	3	–0.0006	0.0013	0.0007	–0.0040	0.0060	0.0020	–0.1325	–0.0155	–0.1480
30	0	–0.0035	–0.0038	–0.0073	–0.0047	–0.0057	–0.0104	–0.0035	–0.0038	–0.0073
	1	–0.0024	0.0000	–0.0024	–0.0055	0.0003	–0.0052	–0.0294	0.0055	–0.0239
	2	–0.0021	0.0005	–0.0016	–0.0065	0.0032	–0.0033	–0.0669	–0.0080	–0.0749
	3	–0.0010	0.0018	0.0008	–0.0040	0.0062	0.0022	–0.1090	–0.0342	–0.1432
60	0	–0.0021	–0.0039	–0.0060	–0.0026	–0.0048	–0.0074	–0.0021	–0.0039	–0.0060
	1	0.0008	–0.0012	–0.0004	–0.0010	0.0000	–0.0010	–0.0195	0.0001	–0.0194
	2	–0.0039	–0.0012	–0.0051	–0.0067	0.0013	–0.0054	–0.0542	–0.0169	–0.0711
	3	0.0001	0.0001	0.0002	–0.0021	0.0027	0.0006	–0.0972	–0.0460	–0.1432

**Table 2:** The error between simulated coverage probability and nominal coverage probability of the 95% two-sided and 97.5% one-sided confidence intervals for the number of groups *G* = 3, one covariate *P* = 1, contrast coefficients {–1, 1/2, 1/2}, error variance σ^2^ = 3.2728, and balanced designs with *N*_*T*_ = 15, 30, and 60.

		Exact approach			Asymptotic method			Direct division		
*N*_*T*_	ψ*	Upper 97.5% CI	Lower 97.5% CI	Two-sided 95% CI	Upper 97.5% CI	Lower 97.5% CI	Two-sided 95% CI	Upper 97.5% CI	Lower 97.5% CI	Two-sided 95% CI
15	0	–0.0009	–0.0023	–0.0032	–0.0032	–0.0041	–0.0073	–0.0009	–0.0023	–0.0032
	1	0.0005	–0.0009	–0.0004	–0.0016	–0.0011	–0.0027	–0.0222	0.0098	–0.0124
	2	0.0002	–0.0002	0.0000	–0.0001	0.0015	0.0014	–0.0655	0.0082	–0.0573
	3	0.0006	0.0004	0.0010	0.0010	0.0025	0.0035	–0.1127	–0.0039	–0.1166
30	0	–0.0008	–0.0005	–0.0013	–0.0023	–0.0014	–0.0037	–0.0008	–0.0005	–0.0013
	1	0.0005	–0.0003	0.0002	–0.0012	0.0001	–0.0011	–0.0210	0.0049	–0.0161
	2	–0.0017	–0.0018	–0.0035	–0.0023	0.0002	–0.0021	–0.0536	–0.0035	–0.0571
	3	0.0011	0.0027	0.0038	0.0005	0.0045	0.0050	–0.0928	–0.0197	–0.1125
60	0	–0.0025	–0.0027	–0.0052	–0.0028	–0.0033	–0.0061	–0.0024	–0.0027	–0.0051
	1	0.0015	–0.0012	0.0003	0.0006	–0.0010	–0.0004	–0.0129	0.0003	–0.0126
	2	–0.0030	0.0003	–0.0027	–0.0041	0.0011	–0.0030	–0.0457	–0.0089	–0.0546
	3	0.0010	0.0013	0.0023	0.0003	0.0033	0.0036	–0.0821	–0.0314	–0.1135

The simulated coverage performance in Tables 1 and 2 suggest that the exact approach is excellent in attaining the nominal confidence levels for all one- and two-sided situations. Although the sample size configurations are not large, the asymptotic method provides surprisingly accurate results and is only slightly inferior to the exact procedure. Note that the small discrepancies of the exact and asymptotic procedures are generally within the 95% simulation variability of 0.006, 0.004, and 0.003 for the nominal coverage probability of 0.90, 0.95 and 0.975, respectively. However, the simulated coverage rates of the direct division are systematically lower than the nominal level, especially for large ψ* > 1. The exact interval procedure generally yields asymmetric confidence intervals for ψ*. Consequently, the equidistant confidence limits of the direct division technique tend to have undesirable effects. For the model configurations examined here, the lower confidence limits of the one-sided upper 95% and 97.5% confidence intervals are substantially less accurate than the counterparts or upper confidence limits of the one-sided lower 95% and 97.5% confidence intervals. The only exceptions that the direct division gives acceptable coverage performance are those associated with ψ* = 0. Note that the three confidence intervals for the standardized contrast effect are asymptotically equivalent. However, when the magnitude is not zero, the direct method is substantially less accurate than the other two procedures even for large sample sizes. In view of these findings, the exact confidence interval estimation is recommended. When no statistical software is available, the asymptotic method provides a viable alternative for hand computation. However, the direct division procedure is too simple to be useful and is not appropriate for general applications. Additional assessments were conducted to demonstrate the intrinsic implications and the results suggest the same performance situations as reported here.

## Results

### Precision assessments and sample size determinations

Within the context of ANCOVA, Hochberg and Varon-Salomon [38] showed that the conditional (on the covariates) confidence interval procedure compares favorably with the unconditional method based on the joint distribution of the response and covariate variables. Thus, the inferential procedures of hypothesis testing and interval estimation are the same under both fixed and random formulations. The distinction between the two modeling setups, however, becomes crucial when testing power, coverage probability, and corresponding sample size calculations are to be made. To exhibit the unique and distinct precision characteristics of confidence intervals, two useful criteria have been considered in Kupper and Hafner [39] for one- and two-sample problems. The suggested precision assessments maintain the expected magnitude of interval widths and the assurance probability of interval widths within a pre-assigned threshold.

### Precision assessments

When planning and conducting ANCOVA research, the actual values of the continuous measurements of response and covariate variables for each subject are available only after the observations are obtained. In addition to the randomness of normal responses, the stochastic nature of covariate variables has to be taken into account in precision analysis under the general and unconditional context of ANCOVA. It is convenient and useful to consider the covariate variables have the independent multinormal distributions

$${\text{X}}_{ij}\sim N_{P}\left(\mu_{Xi},\;\Sigma_{X}\right)$$ (17)

where **μ**_*Xi*_ is a *P* × 1 vector and **Σ**_*X*_ is a *P* × *P* positive-definite variance-covariance matrix for *i* = 1,…, *G*, and *j* = 1, …, *N*_*i*_. The quantity *V* in $Var\left(\hat{\psi }\right)=\sigma^{2}V$ can also be expressed as *V* = *a*(1 + *bF*_*X*_), where $a=\sum_{i=1}^{G}c_{i}^{2}/N_{i}$, *b* = *P*/(ν + 1), and *F*_*X*_ = *Q*/{*ab*(*N*_*T*_−*G*)}. The multivariate results in Anderson [40, Section 5.2.2] and Muirhead [41, Section 3.2.3] show that *F*_*X*_ has the distribution

$$F_{X}\sim F(P,\;\nu +1,\;\xi ),$$ (18)

where *F*(*P*, ν + 1, ξ) is the noncentral *F* distribution with degrees of freedom *P* and ν + 1 and noncentrality parameter ${\xi =(\sum_{i=1}^{G}c_{i}\mu_{Xi})}^{\text{T}}{\left(a\Sigma_{X}\right)}^{-1}(\sum_{i=1}^{G}c_{i}\mu_{Xi})$. It is noted in Anderson [40] that for large samples the distribution of *F*_*X*_ given by Eq 18 is approximately valid even if the parent distribution of covariates is not normal. In addition, the robust features of the *F* and Hotelling’s statistics have been examined empirically and theoretically in Chase and Bulgren [42], Everitt [43], Holloway and Dunn [44], Hopkins and Clay [45], and Kariya [46,47]. Hence, the noncentral *F* distribution provides a robust approximation for general use.

A useful population covariate disparity index can be defined as ${\theta =(\sum_{i=1}^{G}c_{i}\mu_{Xi})}^{\text{T}}\Sigma_{X}^{-1}(\sum_{i=1}^{G}c_{i}\mu_{Xi})$. However, it is a common assumption for a randomized design that the covariate characteristics are identical for all treatment groups with **μ**_*X*1_ = … = **μ**_*XG*_ = **μ**_*X*_, and the noncentrality parameter and covariate disparity reduce to ξ = θ = 0. Accordingly, the scaled quantity *f*^2^* = ξ/*N*_*T*_ = θ/(*aN*_*T*_) = θ/a* has a similar role as the signal to noise ratio *f*^2^ in ANOVA where $a*=\sum_{i=1}^{G}c_{i}^{2}/p_{i}$ and *p*_*i*_ = *N*_*i*_/*N*_*T*_ for *i* = 1, …, *G*. Thus, the conventional benchmark of Cohen [2] for small, medium, and large effects of *f* = 0.10, 0.25, and 0.40 may serve as a general guideline for the magnitude of covariate disparity when no better basis is available. However, the merit of a specific size of disparity should be assessed and interpreted by directly comparing with the findings in related prior studies.

In order to accommodate the stochastic properties of the covariate and response variables through the joint distribution of *F*_*X*_ and T*, the expected width is evaluated for the interval width $W={\hat{\psi }}_{EU}^{*}–{\hat{\psi }}_{EL}^{*}$ as

$$\Omega =E\left[W\right]=E_{F}E_{T}\left[W\right],$$ (19)

where *E*_*F*_[*·*] and *E*_*T*_[*·*] are taken with respect to the noncentral *F* distribution of *F*_*X*_ and the noncentral *t* distribution of T*, respectively. An alternative approach to precision appraisal of confidence intervals is the assurance probability of interval width *W* is not wider than the designated bound ω (> 0):

$$\Gamma =P\{W\leq \omega \}=E_{F}E_{T}\left[G\right],$$ (20)

where *G* = 1 if *W* ≤ ω and *G* = 0 if *W* > ω.

### Sample size determinations

In planning research design, optimal sample size determinations need to be conducted to achieve the designated precision requirement in interval estimation. For the current problem of precise interval estimation of standardized contrasts, it is desirable to determine the minimal sample sizes such that the expected width of a 100(1 − α)% confidence interval is within the selected bound ω (> 0):

Ω≤ω. (21)

Also, one may compute the minimal sample sizes needed to guarantee, with a given assurance probability 1 − γ (< 1), that the interval width of a 100(1 − α)% confidence interval will not exceed the chosen threshold ω:

Γ≥1-γ. (22)

Note that Lai and Kelley [18] presented approximate sample size formulas for interval estimation of standardized contrasts for balanced ANCOVA with one covariate (*N*_*i*_ = *N* and *P* = 1). They assumed that, under a randomized design, the linear contrast of covariate means $\sum_{i=1}^{G}c_{i}{\overset{-}{X}}_{i}$ is usually close to zero and the sample variance of a linear contrast can be approximated as τ^2^ ≐ *a*σ^2^. Extending such a simplification to the general case of ANCOVA designs with one or more covariates, the distribution of $T^{*}=\hat{\psi }/\hat{\tau }$ given in Eq 10 is simplified as

$$T^{*}\dot{\sim }t(\nu ,{\;\lambda }_{a}),$$ (23)

where λ_*a*_ = ψ*/*a*^1/2^. The expected width and assurance probability of interval widths are modified as

$$\Omega_{a}=E_{Ta}\left[W\right]\;\text{and}\;\Gamma_{a}=E_{Ta}\left[G\right],$$ (24)

respectively, where *E*_*Ta*_[*·*] is taken with respect to the approximate noncentral *t* distribution *t*(ν, λ_*a*_) of T*. These two simplified precision functions Ω_*a*_ and Γ_*a*_ provide alternative sample size calculations for standardized contrast analysis. For comparative purposes, these approximate methods are also investigated to reveal the impact of omitted covariate effects on sample size determinations.

### Numerical illustrations

The underlying behavior and relative performance of the exact and approximate procedures for precision and sample size calculations are demonstrated in the following empirical study. First, the minimum sample sizes for the expected width and assurance probability criteria are computed by the exact and approximate distribution functions under the designated model configurations. Second, the accuracy of the precision and sample size procedures is examined through Monte Carlo simulation study to clarify the influence of covariate features under a wide range of model scenarios.

Accordingly, the balanced ANCOVA models with three treatment groups *G* = 3, and one to five (*P* = 1, …, 5) normal covariate variables are used as the basis for numerical assessments. The model configurations have the designated settings: the intercept parameters {μ_1_, μ_2_, μ_3_} = {0.5, 0, 0}, contrast coefficients {*c*_1_, *c*_2_, *c*_3_} = {1, –0.5, –0.5}, and error variance σ^2^ = 1. Note that the resulting standardized contrast effect is ψ* = 0.5. Because the covariate distribution function *F*_*X*_ depends on the group mean values and variance-covariance matrix of the multinormal covariate distributions through the covariate disparity index θ. Without loss of generality, the corresponding mean values are set as **μ**_*X*1_ = {θ^1/2^, 0, …, 0}, **μ**_*X*2_ = **μ**_*X*3_ = **0** where **0** is a *P* × 1 null column vector, and **Σ**_*X*_ = **I**_*P*_ is the identity matrix of dimension *P*. Three different magnitudes of covariate disparity are considered: θ = 0, 0.25 and 0.50 to represent potential covariate characteristics in randomized and nonrandomized designs. Throughout this numerical demonstration, only balanced designs with sample size ratios {*r*_1_, *r*_2_, *r*_3_} = {1, 1, 1} are considered and the confidence level is fixed as 1 –α = 0.95. The computed total sample sizes for homogeneous covariates θ = 0 with the expected width threshold ω = 1.00, 1.25 and 1.50, and *P* = 1, …, 5 are presented in Table 3, while the corresponding total sample sizes associated with θ = 0.25 and 0.50 are summarized in Tables 4 and 5, respectively. The optimal total sample sizes for assurance probability 1 –γ = 0.80 with ω = 1.00, 1.25 and 1.50 of bounded confidence intervals are summarized in Tables 6–8 for θ = 0, 0.25 and 0.50, respectively.

**Table 3:** Computed sample size, estimated expected width, and simulated expected width for 95% confidence interval of standardized contrast when the number of groups *G* = 3, standardized contrast effect ψ* = 0.5, and covariate disparity θ = 0.

		The proposed approach				The approximate method			
ω	Number of covariate(s)	Total sample size	Estimated expected width	Simulated expected width	Difference	Total sample size	Estimated expected width	Simulated expected width	Difference
1.00	1	75	0.9845	0.9844	0.0001	72	0.9982	1.0053	–0.0071
	2	75	0.9912	0.9914	–0.0002	72	0.9984	1.0129	–0.0145
	3	75	0.9932	0.9989	–0.0058	72	0.9987	1.0204	–0.0217
	4	78	0.9854	0.9854	0.0000	72	0.9990	1.0288	–0.0298
	5	78	0.9927	0.9926	0.0001	72	0.9993	1.0368	–0.0375
1.25	1	48	1.2392	1.2392	0.0000	48	1.2257	1.2393	–0.0136
	2	51	1.2139	1.2140	–0.0001	48	1.2263	1.2540	–0.0276
	3	51	1.2241	1.2275	–0.0034	48	1.2269	1.2687	–0.0418
	4	51	1.2420	1.2417	0.0003	48	1.2276	1.2853	–0.0577
	5	54	1.2166	1.2177	–0.0011	48	1.2283	1.3018	–0.0735
1.50	1	36	1.4410	1.4411	–0.0001	33	1.4841	1.5091	–0.0250
	2	36	1.4646	1.4645	0.0001	33	1.4854	1.5359	–0.0505
	3	36	1.4875	1.4896	–0.0021	33	1.4869	1.5663	–0.0794
	4	39	1.4468	1.4461	0.0007	33	1.4885	1.5968	–0.1083
	5	39	1.4708	1.4706	0.0001	33	1.4902	1.6309	–0.1407

**Table 4:** Computed sample size, estimated expected width, and simulated expected width for 95% confidence interval of standardized contrast when the number of groups *G* = 3, standardized contrast effect ψ* = 0.5, and covariate disparity θ = 0.25.

		The proposed approach				The approximate method			
ω	Number of covariate(s)	Total sample size	Estimated expected width	Simulated expected width	Difference	Total sample size	Estimated expected width	Simulated expected width	Difference
1.00	1	78	0.9916	0.9915	0.0001	72	0.9982	1.0332	–0.0350
	2	78	0.9985	0.9982	0.0003	72	0.9984	1.0407	–0.0422
	3	81	0.9851	0.9856	–0.0005	72	0.9987	1.0484	–0.0497
	4	81	0.9925	0.9922	0.0003	72	0.9990	1.0573	–0.0583
	5	81	0.9994	0.9989	0.0004	72	0.9993	1.0655	–0.0662
1.25	1	51	1.2348	1.2345	0.0003	48	1.2257	1.2750	–0.0493
	2	51	1.2484	1.2484	0.0000	48	1.2263	1.2895	–0.0632
	3	54	1.2231	1.2232	–0.0001	48	1.2269	1.3055	–0.0785
	4	54	1.2371	1.2362	0.0009	48	1.2276	1.3221	–0.0945
	5	57	1.2131	1.2134	–0.0003	48	1.2283	1.3382	–0.1099
1.50	1	36	1.4828	1.4830	–0.0002	33	1.4841	1.5526	–0.0685
	2	39	1.4426	1.4426	0.0000	33	1.4854	1.5800	–0.0945
	3	39	1.4642	1.4648	–0.0006	33	1.4869	1.6127	–0.1258
	4	39	1.4885	1.4879	0.0006	33	1.4885	1.6435	–0.1551
	5	42	1.4474	1.4486	–0.0013	33	1.4902	1.6783	–0.1881

**Table 5:** Computed sample size, estimated expected width, and simulated expected width for 95% confidence interval of standardized contrast when the number of groups *G* = 3, standardized contrast effect ψ* = 0.5, and covariate disparity θ = 0.50.

		The proposed approach				The approximate method			
ω	Number of covariate(s)	Total sample size	Estimated expected width	Simulated expected width	Difference	Total sample size	Estimated expected width	Simulated expected width	Difference
1.00	1	81	0.9982	0.9975	0.0007	72	0.9982	1.0604	–0.0622
	2	84	0.9860	0.9867	–0.0007	72	0.9984	1.0680	–0.0695
	3	84	0.9923	0.9931	–0.0008	72	0.9987	1.0760	–0.0773
	4	84	0.9989	0.9987	0.0002	72	0.9990	1.0852	–0.0862
	5	87	0.9867	0.9862	0.0005	72	0.9993	1.0938	–0.0945
1.25	1	54	1.2306	1.2308	–0.0002	48	1.2257	1.3095	–0.0838
	2	54	1.2433	1.2421	0.0012	48	1.2263	1.3243	–0.0979
	3	57	1.2201	1.2213	–0.0012	48	1.2269	1.3407	–0.1138
	4	57	1.2327	1.2324	0.0003	48	1.2276	1.3577	–0.1301
	5	57	1.2454	1.2458	–0.0004	48	1.2283	1.3740	–0.1457
1.50	1	39	1.4599	1.4588	0.0010	33	1.4841	1.5952	–0.1112
	2	39	1.4820	1.4821	–0.0001	33	1.4854	1.6235	–0.1381
	3	42	1.4431	1.4428	0.0003	33	1.4869	1.6576	–0.1707
	4	42	1.4640	1.4631	0.0009	33	1.4885	1.6891	–0.2006
	5	42	1.4866	1.4881	–0.0014	33	1.4902	1.7246	–0.2345

**Table 6:** Computed sample size, estimated assurance probability, and simulated assurance probability for 95% confidence interval of standardized contrast when the number of groups *G* = 3, standardized contrast effect ψ* = 0.5, assurance probability 1 –γ = 0.80, and covariate disparity θ = 0.

		The proposed approach				The approximate method			
ω	Number of covariate(s)	Total sample size	Estimated assurance probability	Simulated assurance probability	Difference	Total sample size	Estimated assurance probability	Simulated assurance probability	Difference
1.00	1	75	0.8293	0.8299	–0.0006	75	0.9114	0.8312	0.0802
	2	78	0.9125	0.9128	–0.0003	75	0.9121	0.7145	0.1976
	3	78	0.8420	0.8358	–0.0118	75	0.9082	0.5980	0.3102
	4	81	0.9186	0.9214	–0.0028	75	0.8992	0.4761	0.4231
	5	81	0.8686	0.8680	0.0006	75	0.9000	0.3540	0.5460
1.25	1	51	0.9312	0.9297	0.0015	48	0.8479	0.7135	0.1344
	2	51	0.8572	0.8586	–0.0014	48	0.8432	0.5510	0.2922
	3	54	0.9240	0.9312	–0.0072	48	0.8309	0.4012	0.4297
	4	54	0.8715	0.8709	0.0006	48	0.8337	0.2591	0.5746
	5	57	0.9324	0.9278	0.0046	48	0.8290	0.1671	0.6619
1.50	1	36	0.8898	0.8830	0.0068	36	0.9604	0.8903	0.0701
	2	39	0.9451	0.9451	0.0000	36	0.9542	0.7751	0.1791
	3	39	0.8868	0.8841	0.0027	36	0.9473	0.6468	0.3005
	4	39	0.8084	0.8084	0.0000	36	0.9464	0.4969	0.4495
	5	42	0.9001	0.8980	0.0021	36	0.9388	0.3710	0.5678

**Table 7:** Computed sample size, estimated assurance probability, and simulated assurance probability for 95% confidence interval of standardized contrast when the number of groups *G* = 3, standardized contrast effect ψ* = 0.5, assurance probability 1 –γ = 0.80, and covariate disparity θ = 0.25.

		The proposed approach				The approximate method			
ω	Number of covariate(s)	Total sample size	Estimated assurance probability	Simulated assurance probability	Difference	Total sample size	Estimated assurance probability	Simulated assurance probability	Difference
1.00	1	81	0.8345	0.8271	0.0074	75	0.9114	0.4243	0.4871
	2	84	0.8948	0.8932	0.0016	75	0.9121	0.3300	0.5821
	3	84	0.8560	0.8571	–0.0011	75	0.9082	0.2468	0.6614
	4	84	0.8101	0.8139	–0.0038	75	0.8992	0.1776	0.7216
	5	87	0.8767	0.8805	–0.0038	75	0.9000	0.1239	0.7761
1.25	1	54	0.8661	0.8623	0.0038	48	0.8479	0.3882	0.4597
	2	54	0.8070	0.8093	–0.0023	48	0.8432	0.2874	0.5558
	3	57	0.8816	0.8795	0.0021	48	0.8309	0.1917	0.6392
	4	57	0.8340	0.8371	–0.0031	48	0.8337	0.1162	0.7175
	5	60	0.8995	0.8973	0.0022	48	0.8290	0.0693	0.7597
1.50	1	39	0.8740	0.8713	0.0027	36	0.9604	0.6804	0.2800
	2	39	0.8066	0.8103	–0.0037	36	0.9542	0.5581	0.3961
	3	42	0.8874	0.8921	–0.0047	36	0.9473	0.4390	0.5083
	4	42	0.8337	0.8360	–0.0023	36	0.9464	0.3165	0.6299
	5	45	0.9042	0.9072	–0.0030	36	0.9388	0.2251	0.7137

**Table 8:** Computed sample size, estimated assurance probability, and simulated assurance probability for 95% confidence interval of standardized contrast when the number of groups *G* = 3, standardized contrast effect ψ* = 0.5, assurance probability 1 –γ = 0.80, and covariate disparity θ = 0.50.

		The proposed approach				The approximate method			
ω	Number of covariate(s)	Total sample size	Estimated assurance probability	Simulated assurance probability	Difference	Total sample size	Estimated assurance probability	Simulated assurance probability	Difference
1.00	1	87	0.8576	0.8571	0.0005	75	0.9114	0.1765	0.7349
	2	87	0.8194	0.8162	0.0032	75	0.9121	0.1285	0.7836
	3	90	0.8766	0.8808	–0.0042	75	0.9082	0.0880	0.8202
	4	90	0.8437	0.8411	0.0026	75	0.8992	0.0595	0.8397
	5	90	0.8052	0.8080	–0.0028	75	0.9000	0.0395	0.8605
1.25	1	57	0.8382	0.8447	–0.0065	48	0.8479	0.1986	0.6493
	2	60	0.8867	0.9009	–0.0042	48	0.8432	0.1340	0.7092
	3	60	0.8608	0.8651	–0.0043	48	0.8309	0.0920	0.7389
	4	60	0.8167	0.8187	–0.0020	48	0.8337	0.0536	0.7801
	5	63	0.8817	0.8859	–0.0042	48	0.8290	0.0276	0.8014
1.50	1	42	0.8800	0.8781	0.0019	36	0.9604	0.4908	0.4696
	2	42	0.8308	0.8314	–0.0006	36	0.9542	0.3787	0.5755
	3	45	0.8972	0.8957	0.0015	36	0.9473	0.2845	0.6628
	4	45	0.8560	0.8645	–0.0085	36	0.9464	0.1956	0.7508
	5	45	0.8033	0.8100	–0.0067	36	0.9388	0.1369	0.8019

It can be seen from the results of the proposed approach in Tables 3–8 that larger sample sizes are required for smaller expected width threshold ω and the results for ω = 1 are almost twice as those of ω = 1.5 in the same table. Also, the assurance probability principle demands larger sample sizes than the expected width criterion for the settings examined here. It is expected that the necessary sample sizes will increase for higher assurance probability levels 1 –γ > 0.80. The computed sample sizes of the approximate method also have similar patterns with respect to the width threshold and precision criteria. However, because the omission of covariate features, the computed sample sizes do not vary with the number of covariates *P* and the covariate disparity θ. The estimated expected width and estimated assurance probability of the exact and approximate procedures are also listed in Tables 3–8.

In the second stage, simulated values of expected width and assurance probability associated with the reported sample sizes and selected parameter configurations are computed through a Monte Carlo study of 10,000 independent data sets. For each replicate, *N*_*T*_ sets of covariate values are generated from the prescribed multinormal distribution. These values of covariates, treatment effects, and error variance σ^2^, in turn, determine the mean responses for generating *N*_*T*_ normal outcomes of the ANCOVA model. Because the covariate coefficients are nuisance parameters, they are set as β_1_ = … = β_*P*_ = 0.5 in the precision analysis. According to the differences between the estimated and simulated precision quantities of Ω and Γ in Tables 3–8, the exact approach results in outstanding performance for all 90 cases. In contrast, the approximate method does not provide accurate sample size calculations for most of the cases. Note that the simplified precision assessments Ω_*a*_ and Γ_*a*_ are presumed valid under the assumption of randomized designs, such as the situations with covariate disparity θ = 0 in Tables 3 and 6. However, the estimated expected width Ω_*a*_ is consistently less than the simulated expected width for all the cases in Table 3. The results in Table 6 reveal that the assurance probability calculation Γ_*a*_ is substantially larger than the simulated assurance probability. Therefore, the approximate method generally underestimates the sample sizes and the deficiency phenomenon is more prominent and noticeably problematic for the cases with covariate disparity θ = 0.25 and 0.50 in Tables 4, 5, 7 and 8. Consequently, the usefulness of the approximate procedures is extremely limited and the exact techniques are recommended for precision assessments and sample size determinations. Additional numerical assessments were also conducted for non-normal error *t*(10) and non-normal covariates: Exponential(1), Gamma(5, 5^−1/2^), Laplace(0, 1), Log normal(0, 1/4), and Uniform(0, 1). The results show that the precision outcomes for expected width and assurance probability are marginally affected by the violations of normal assumption. Overall, the suggested confidence interval and sample size procedures preserve reasonably good performance under various non-normal error and covariate situations. To facilitate the execution of the suggested sample size procedures, SAS/IML and R computer algorithms are presented in the supplemental file. For example, the user specifications of the supplemental SAS file B for performing sample size calculations to obtain designated expected width are as follows:

PROC IML;
*USER SPECIFICATION PORTION;
*DESIGNATED ALPHA;ALPHA = 0.05;
*NUMBER OF GROUPS;G = 3;
*NUMBER OF COVARIATES;P = 5;
*VARIANCE;SIGSQ = 1;
*GROUP RATIOS;RVEC = J(1,G,1);
*CONTRAST COEFFICIENTS;CVEC = {1–0.5–0.5};
*STANDADIZED CONTRAST;PSIS = 0.5;
*COVARIATE DISPARITY;THETA = 0;
*DESIGNATED WIDTH;OMEGA = 1.5;
*END OF USER SPECIFICATION PORTION;

Moreover, the user specifications of the supplemental SAS file C, for performing sample size calculations to ensure adequate assurance probability of achieving the desired width, are listed as

PROC IML;
*USER SPECIFICATION PORTION;
*DESIGNATED ALPHA;ALPHA = 0.05;
*NUMBER OF GROUPS;G = 3;
*NUMBER OF COVARIATES;P = 5;
*VARIANCE;SIGSQ = 1;
*GROUP RATIOS;RVEC = J(1,G,1);
*CONTRAST COEFFICIENTS;CVEC = {1–0.5–0.5};
*STANDADIZED CONTRAST;PSIS = 0.5;
*COVARIATE DISPARITY;THETA = 0;
*DESIGNATED WIDTH;OMEGA = 1.5;
*ASSURANCE PROBABILITY;AP = 0.80;
*END OF USER SPECIFICATION PORTION;

The corresponding specifications of R programs for expected width and assurance probability are presented in supplemental files E and F, respectively:

ancova.scie.apx2.fun(alpha = 0.05, g = 3, p = 1, sigsq = 1, rvec = c(1,1,1), cvec = c(1,-0.5,-0.5), psis = 0.5, theta = 0, omega = 1.5)

and

ancova.scie.apx3.fun(alpha = 0.05, g = 3, p = 1, sigsq = 1, rvec = c(1,1,1), cvec = c(1,-0.5,-0.5), psis = 0.5, theta = 0, omega = 1.5, ap = 0.8).

It should be emphasized that the analytic complexity requires computer algorithms to compute the exact confidence intervals and optimal sample sizes under various design configurations. The developed computer programs substantially facilitate the recommended confidence interval and sample size techniques in practical applications. Users can easily alter the exemplifying settings in the programs with their designated model specifications.

## Conclusions

Measures of effect size and confidence intervals are extremely useful for comparing quantitative information across different studies. This research describes and compares exact and approximate confidence intervals for standardized contrast effects in ANCOVA. The theoretical and numerical findings show that the exact approach has analytic support and empirical advantage over the other two approximate methods using asymptotic theory and direct division. The advanced aspects of precision appraisals and sample size calculations are also investigated for interval estimation of standardized contrasts. The existing study provided a shortcut under the speculation that covariate disparity is unlikely to exist in randomized designs. However, the approximate technique actually does not give reliable precision and sample size outcomes even when there is no covariate disparity. The proposed exact approach has the distinct utility of accommodating the full distributional information of normal covariates, especially the potential disparity of unequal means for both random and nonrandom ANCOVA designs. Overall, the described results of interval estimation, precision assessment, and sample size planning for standardized contrasts update and expand upon current work of ANCOVA in the literature.

## Supporting information

S1 File SAS programs for standardized contrast effects in ANCOVA designs. (PDF)

S2 File R programs for standardized contrast effects in ANCOVA designs. (PDF)
