# Supplementary material for: Assessing standardized contrast effects in ANCOVA: Confidence intervals, precision evaluations, and sample size requirements
Source: PLoS One. 2023 Feb 24;18(2):e0282161. doi: 10.1371/journal.pone.0282161 (PMC9955653; doi:10.1371/journal.pone.0282161)
Supplement: S1 File — (PDF) [file pone.0282161.s001.pdf]

Supplemental file A

SAS IML program for computing confidence interval of standardized contrast

```
PROC IML;
*USER SPECIFICATION PORTION;
*DESIGNATED ALPHA;ALPHA=0.10;
*NUMBER OF GROUPS;G=3;
*NUMBER OF COVARIATES;P=1;
*TOAL SAMPLE SIZE;NT=59;
*CONTRAST ESTIMATE;PSIH=2.4823;
*SAMPLE VARIANCE;SIGSQH=3.2728;
*FACTOR FOR THE VARIANCE OF A LINEAR CONTRAST TAUSQ;V=0.081437;
*END OF USER SPECIFICATION PORTION;

PSISH=PSIH/SQRT(SIGSQH);
TAUSQH=SIGSQH#V;
DF=NT-G-P;
TS=PSIH/SQRT(TAUSQH);
U=GAMMA(DF/2)/(SQRT(DF/2)#GAMMA((DF-1)/2));
PSISH_UB=U#PSISH;
COVERP=1-ALPHA;
PSISHL=TNONCT(TS,DF,1-ALPHA/2)#SQRT(V);
PSISHU=TNONCT(TS,DF,ALPHA/2)#SQRT(V);
PRINT TS[FORMAT=8.4] PSISH[FORMAT=8.4] PSISH_UB[FORMAT=8.4];
PRINT 'CI FOR PSIS' ALPHA COVERP PSISHL[FORMAT=8.4]
PSISHU[FORMAT=8.4];
QUIT;
```

Supplemental file B

SAS IML program for performing sample size calculations to obtain designated expected width for confidence interval of standardized contrast

```
PROC IML;
*USER SPECIFICATION PORTION;
*DESIGNATED ALPHA;ALPHA=0.05;
*NUMBER OF GROUPS;G=3;
*NUMBER OF COVARIATES;P=5;
*VARIANCE;SIGSQ=1;
*GROUP RATIOS;RVEC=J(1,G,1);
*CONTRAST COEFFICIENTS;CVEC={1 -0.5 -0.5};
*STANDADIZED CONTRAST;PSIS=0.5;
*COVARIATE DISPARITY;THETA=0;
*DESIGNATED WIDTH;OMEGA=1.5;
*END OF USER SPECIFICATION PORTION;

PRINT ALPHA G P PSIS THETA OMEGA;
PRINT RVEC CVEC;
ALPHAL=ALPHA/2;ALPHAU=1-ALPHA/2;
NUMINT=100;L=NUMINT+1;DD=1E-5;
COEVEC=({1}||REPEAT({4 2},1,NUMINT/2-1)||{4 1})`;
BL=DD;BU=1-DD;INTBL=(BU-BL)/NUMINT;BVEC=BL+INTBL#(0:NUMINT)`;
START FINDTBEW;QUAN=J(L,1,0);
NVEC=N#RVEC;NT=SUM(NVEC);DF=NT-G-P;A=SUM((CVEC##2)/NVEC);DFX=D
F+1;B=P/DFX;
IF P=1 THEN DO;
TXL=QUANTILE('T',DD,DFX,SQRT(THETA/A));
TXU=QUANTILE('T',1-DD,DFX,SQRT(THETA/A));
INTTXL=(TXU-TXL)/NUMINT;TXVEC=TXL+INTTXL#(0:NUMINT)`;
WTXPDF=(INTTXL/3)#COEVEC#PDF('T',TXVEC,DFX,SQRT(THETA/A));
ABVEC=SQRT(A#(1+B#TXVEC##2));
DO I=1 TO L;
ABVECI=ABVEC[I,1];
NCPI=PSIS/ABVECI;
LT=TINV(DD,DF,NCPI);UT=TINV(1-DD,DF,NCPI);
INTLT=(UT-LT)/NUMINT;TVEC=LT+INTLT#(0:NUMINT)`;
WTPDF=(INTLT/3)#COEVEC#PDF('T',TVEC,DF,NCPI);
```

```
PSISL=TNONCT(TVEC,DF,ALPHAU)#ABVECI;
PSISU=TNONCT(TVEC,DF,ALPHAL)#ABVECI;
WVEC=PSISU-PSISL;
QUAN[I,1]=WTPDF`*WVEC;
END;
EEW=WTXPDF`*QUAN;
END;ELSE DO;
FVEC=BVEC/(B#(1-BVEC));
BPDF=PDF('F',FVEC,P,DFX,THETA/A)/(B#(1-BVEC)##2);
WBPDF=(INTBL/3)#COEVEC#BPDF;
ABVEC=SQRT(A#(1+B#FVEC));
DO I=1 TO L;
ABVECI=ABVEC[I,1];
NCPI=PSIS/ABVECI;
LT=TINV(DD,DF,NCPI);UT=TINV(1-DD,DF,NCPI);
INTLT=(UT-LT)/NUMINT;TVEC=LT+INTLT#(0:NUMINT)`;
WTPDF=(INTLT/3)#COEVEC#PDF('T',TVEC,DF,NCPI);
PSISL=TNONCT(TVEC,DF,ALPHAU)#ABVECI;
PSISU=TNONCT(TVEC,DF,ALPHAL)#ABVECI;
WVEC=PSISU-PSISL;
QUAN[I,1]=WTPDF`*WVEC;
END;
EEW=WBPDF`*QUAN;
END;
FINISH;
N=9;LOOP=0;
DO UNTIL (EEW<OMEGA | LOOP>1000);
N=N+1;LOOP=LOOP+1;
RUN FINDTBEW;END;
PRINT NVEC NT EEW[FORMAT=8.4];
QUIT;
```

Supplemental file C

SAS IML program for performing sample size calculations to ensure adequate assurance probability of achieving the desired width for confidence interval of standardized contrast

```
PROC IML;
*USER SPECIFICATION PORTION;
*DESIGNATED ALPHA;ALPHA=0.05;
*NUMBER OF GROUPS;G=3;
*NUMBER OF COVARIATES;P=5;
*VARIANCE;SIGSQ=1;
*GROUP RATIOS;RVEC=J(1,G,1);
*CONTRAST COEFFICIENTS;CVEC={1 -0.5 -0.5};
*STANDADIZED CONTRAST;PSIS=0.5;
*COVARIATE DISPARITY;THETA=0;
*DESIGNATED WIDTH;OMEGA=1.5;
*ASSURANCE PROBABILITY;AP=0.80;
*END OF USER SPECIFICATION PORTION;

PRINT ALPHA G P PSIS THETA OMEGA AP;
PRINT RVEC CVEC;
ALPHAL=ALPHA/2;ALPHAU=1-ALPHA/2;
NUMINT=100;L=NUMINT+1;DD=1E-5;
COEVEC=({1}||REPEAT({4 2},1,NUMINT/2-1)||{4 1})`;
BL=DD;BU=1-DD;INTBL=(BU-BL)/NUMINT;BVEC=BL+INTBL#(0:NUMINT)`;
START FINDTBAP;QUAN=J(L,1,0);
NVEC=N#RVEC;NT=SUM(NVEC);DF=NT-G-P;A=SUM((CVEC##2)/NVEC);DFX=D
F+1;B=P/DFX;
IF P=1 THEN DO;
TXL=QUANTILE('T',DD,DFX,SQRT(THETA/A));
TXU=QUANTILE('T',1-DD,DFX,SQRT(THETA/A));
INTTXL=(TXU-TXL)/NUMINT;TXVEC=TXL+INTTXL#(0:NUMINT)`;
WTXPDF=(INTTXL/3)#COEVEC#PDF('T',TXVEC,DFX,SQRT(THETA/A));
ABVEC=SQRT(A#(1+B#TXVEC##2));
DO I=1 TO L;
ABVECI=ABVEC[I,1];
NCPI=PSIS/ABVECI;
LT=TINV(DD,DF,NCPI);UT=TINV(1-DD,DF,NCPI);
INTLT=(UT-LT)/NUMINT;TVEC=LT+INTLT#(0:NUMINT)`;
```

```
WTPDF=(INTLT/3)#COEVEC#PDF('T',TVEC,DF,NCPI);
PSISL=TNONCT(TVEC,DF,ALPHAU)#ABVECI;
PSISU=TNONCT(TVEC,DF,ALPHAL)#ABVECI;
WVEC=PSISU-PSISL;
QUAN[I,1]=WTPDF`*(WVEC<OMEGA);
END;
EAP=WTXPDF`*QUAN;
END;ELSE DO;
FVEC=BVEC/(B#(1-BVEC));
BPDF=PDF('F',FVEC,P,DFX,THETA/A)/(B#(1-BVEC)##2);
WBPDF=(INTBL/3)#COEVEC#BPDF;
ABVEC=SQRT(A#(1+B#FVEC));
DO I=1 TO L;
ABVECI=ABVEC[I,1];
NCPI=PSIS/ABVECI;
LT=TINV(DD,DF,NCPI);UT=TINV(1-DD,DF,NCPI);
INTLT=(UT-LT)/NUMINT;TVEC=LT+INTLT#(0:NUMINT)`;
WTPDF=(INTLT/3)#COEVEC#PDF('T',TVEC,DF,NCPI);
PSISL=TNONCT(TVEC,DF,ALPHAU)#ABVECI;
PSISU=TNONCT(TVEC,DF,ALPHAL)#ABVECI;
WVEC=PSISU-PSISL;
QUAN[I,1]=WTPDF`*(WVEC<OMEGA);
END;
EAP=WBPDF`*QUAN;
END;
FINISH;
N=9;LOOP=0;
DO UNTIL (EAP>AP | LOOP>1000);
N=N+1;LOOP=LOOP+1;
RUN FINDTBAP;END;
PRINT NVEC NT EAP[FORMAT=8.4];
QUIT;
```
