# Supplementary material for: Assessing standardized contrast effects in ANCOVA: Confidence intervals, precision evaluations, and sample size requirements
Source: PLoS One. 2023 Feb 24;18(2):e0282161. doi: 10.1371/journal.pone.0282161 (PMC9955653; doi:10.1371/journal.pone.0282161)
Supplement: S2 File — (PDF) [file pone.0282161.s002.pdf]

Supplemental file D

R program for computing confidence interval of standardized linear contrast

```
ancova.scie.apx1.fun<-function(alpha=0.1, g=3, p=1, nt=59, psih=2.4823,
sigsqh=3.2728, v=0.081437) {
#USER SPECIFICATIONS PORTION
#alpha<-0.10 #DESIGNATED ALPHA
#g<-3 #NUMBER OF GROUPS
#p<-1 #NUMBER OF COVARIATES
#nt<-59 #TOTAL SAMPLE SIZE
#psih<-2.4823 #CONTRAST ESTIMATE
#sigsqh<-3.2728 #SAMPLE VARIANCE
#v<-0.081437 #FACTOR FOR THE VARIANCE OF A LINEAR CONTRAST TAUSQ
#END OF SPECIFICATION

nonct<-function(qu,df,cdf){
dl<--10
du<-10
ecdf<-0
diff<-100
loop<-0
while ((ecdf<cdf | diff>1e-5) & loop<1001){
loop<-loop+1
da<-(dl+du)/2
ecdf<-pt(qu,df,da)
diff<-abs(ecdf-cdf)
if (ecdf>cdf) {dl<-da}
else if (ecdf<cdf) {du<-da}}
return(da)}

psish<-psih/sqrt(sigsqh)
tausqh<-sigsqh*v
df<-nt-g-p
ts<-psih/sqrt(tausqh)
u<-gamma(df/2)/(sqrt(df/2)*gamma((df-1)/2))
psish_ub<-u*psish
coverp<-1-alpha
psishl<-nonct(ts,df,1-alpha/2)*sqrt(v)
psishu<-nonct(ts,df,alpha/2)*sqrt(v)
print("ts, psish, psish_ub")
print(c(ts,psish,psish_ub),digits=5)
print("ci for psis, alpha, coverp, psishl, psishu")
print(c(alpha, coverp, psishl, psishu),digits=4)}
===============
> ancova.scie.apx1.fun(alpha=0.1, g=3, p=1, nt=59, psih=2.4823,
sigsqh=3.2728, v=0.081437)
```

```
[1] "ts, psish, psish_ub"
[1] 4.8082 1.3721 1.3533
[1] "ci for psis, alpha, coverp, psishl, psishu"
[1] 0.1000 0.9000 0.8503 1.8827
```

Supplemental file E

R program for performing sample size calculations to obtain designated expected half-width for confidence interval of standardized contrast

```
ancova.scie.apx2.fun<-function (alpha=0.05, g=3, p=1, sigsq=1,
rvec=c(1,1,1), cvec=c(1,-0.5,-0.5), psis=0.5, theta=0, omega=1.5) {
#USER SPECIFICATIONS PORTION
#alpha<-0.05 #DESIGNATED ALPHA
#g<-3 #NUMBER OF GROUPS
#p<-1 #NUMBER OF COVARIATES
#sigsq<-1 #VARIANCE
#rvec<-c(1,1,1) #GROUP RATIOS
#cvec<-c(1,-0.5,-0.5) #CONTRAST COEFFICIENTS
#psis<-0.5 #STANDARDIZED CONTRAST
#theta<-0 #COVARIATE DISPARITY
#omega<-1.5 #DESIGNATED WIDTH
#END OF SPECIFICATION

tnonct<-function(qu,df,cdf){
dl<--10
du<-10
ecdf<-0
diff<-100
loop<-0
while ((diff<0 | diff>1e-4) & loop<1001){
loop<-loop+1
da<-(dl+du)/2
ecdf<-pt(qu,df,da)
diff<-ecdf-cdf
if (ecdf>cdf) {dl<-da}
else if (ecdf<cdf) {du<-da}}
return(da)}

print("g, p, alpha, psis, theta, omega, rvec, cvec")
print(c(g,p,alpha,psis,theta,omega,rvec,cvec))
alphal<-alpha/2
alphau<-1-alphal
numint<-100
l<-numint+1
dd<-1e-5
coevec<-c(1,rep(c(4,2),numint/2-1),4,1)
bl<-dd
bu<-1-dd
intbl<-(bu-bl)/numint
bvec<-bl+intbl*(0:numint)
```

```
findtbew<-function(){
quan<-rep(0,l)
nvec<-n*rvec
nt<-sum(nvec)
df<-nt-g-p
a<-sum(cvec^2/nvec)
dfx<-df+1
b<-p/dfx
thetaa<-theta/a
if (p==1) {
txl<-qt(dd,dfx,sqrt(thetaa))
txu<-qt(1-dd,dfx,sqrt(thetaa))
inttxl<-(txu-txl)/numint
txvec<-txl+inttxl*(0:numint)
wtxpdf<-(inttxl/3)*coevec*dt(txvec,dfx,sqrt(thetaa))
abvec<-sqrt(a*(1+b*txvec^2))
for (i in seq(l)) {
abveci<-abvec[i]
ncpi<-psis/abveci
lt<-qt(dd,df,ncpi)
ut<-qt(1-dd,df,ncpi)
intlt<-(ut-lt)/numint
tvec<-lt+intlt*(0:numint)
wtpdf<-(intlt/3)*coevec*dt(tvec,df,ncpi)
psisl<-rep(0,l)
psisu<-rep(0,l)
for (j in seq(l)) {
psisl[j]<-tnonct(tvec[j],df,alphau)*abveci
psisu[j]<-tnonct(tvec[j],df,alphal)*abveci}
wvec<-psisu-psisl
quan[i]<-sum(wtpdf*wvec)}
eew<-sum(wtxpdf*quan)}
else {
fvec<-bvec/(b*(1-bvec))
bpdf<-df(fvec,p,dfx,thetaa)/(b*(1-bvec)^2)
wbpdf<-(intbl/3)*coevec*bpdf
abvec<-sqrt(a*(1+b*fvec))
for (i in seq(l)) {
abveci<-abvec[i]
ncpi<-psis/abveci
lt<-qt(dd,df,ncpi)
ut<-qt(1-dd,df,ncpi)
intlt<-(ut-lt)/numint
tvec<-lt+intlt*(0:numint)
wtpdf<-(intlt/3)*coevec*dt(tvec,df,ncpi)
psisl<-rep(0,l)
psisu<-rep(0,l)
```

```
for (j in seq(l)) {
psisl[j]<-tnonct(tvec[j],df,alphau)*abveci
psisu[j]<-tnonct(tvec[j],df,alphal)*abveci}
wvec<-psisu-psisl
quan[i]<-sum(wtpdf*wvec)}
eew<-sum(wbpdf*quan)}}

n<-9
eew<-100
while(eew>omega & n<1001){
n<-n+1
nvec<-n*rvec
nt<-sum(nvec)
eew<-findtbew()}
print("group sizes, nt, expected half-width")
print(c(nvec,nt,eew),digits=6)}
===================
> ancova.scie.apx2.fun(alpha=0.05, g=3, p=1, sigsq=1, rvec=c(1,1,1),
 cvec=c(1,-0.5,-0.5), psis=0.5, theta=0, omega=1.5)

[1] "g, p, alpha, psis, theta, omega, rvec, cvec"
 [1]  3.00  1.00  0.05  0.50  0.00  1.50  1.00  1.00  1.00  1.00 -0.50
 -0.50
[1] "group sizes, nt, expected half-width"
[1] 12.00000 12.00000 12.00000 36.00000  1.44097
```

Supplemental file F

R program for performing sample size calculations to ensure adequate assurance probability of achieving the desired width for confidence interval of standardized contrast

```
ancova.scie.apx3.fun<-function (alpha=0.05, g=3, p=1, sigsq=1,
rvec=c(1,1,1), cvec=c(1,-0.5,-0.5), psis=0.5, theta=0, omega=1.5,
ap=0.8) {
#USER SPECIFICATIONS PORTION
#alpha<-0.05 #DESIGNATED ALPHA
#g<-3 #NUMBER OF GROUPS
#p<-1 #NUMBER OF COVARIATES
#sigsq<-1 #VARIANCE
#rvec<-c(1,1,1) #GROUP RATIOS
#cvec<-c(1,-0.5,-0.5) #CONTRAST COEFFICIENTS
#psis<-0.5 #STANDARDIZED CONTRAST
#theta<-0 #COVARIATE DISPARITY
#omega<-1.5 #DESIGNATED WIDTH
#ap<-0.8 #ASSURANCE PROBABILITY
#END OF SPECIFICATION

tnonct<-function(qu,df,cdf){
dl<--10
du<-10
ecdf<-0
diff<-100
loop<-0
while ((diff<0 | diff>1e-4) & loop<1001){
loop<-loop+1
da<-(dl+du)/2
ecdf<-pt(qu,df,da)
diff<-ecdf-cdf
if (ecdf>cdf) {dl<-da}
else if (ecdf<cdf) {du<-da}}
return(da)}

print("g, p, alpha, psis, theta, omega, ap, rvec, cvec")
print(c(g,p,alpha,psis,theta,omega,ap,rvec,cvec))
alphal<-alpha/2
alphau<-1-alphal
numint<-100
l<-numint+1
dd<-1e-5
coevec<-c(1,rep(c(4,2),numint/2-1),4,1)
bl<-dd
bu<-1-dd
intbl<-(bu-bl)/numint
```

```
bvec<-bl+intbl*(0:numint)

findtbap<-function(){
quan<-rep(0,l)
nvec<-n*rvec
nt<-sum(nvec)
df<-nt-g-p
a<-sum(cvec^2/nvec)
dfx<-df+1
b<-p/dfx
thetaa<-theta/a
if (p==1) {
txl<-qt(dd,dfx,sqrt(thetaa))
txu<-qt(1-dd,dfx,sqrt(thetaa))
inttxl<-(txu-txl)/numint
txvec<-txl+inttxl*(0:numint)
wtxpdf<-(inttxl/3)*coevec*dt(txvec,dfx,sqrt(thetaa))
abvec<-sqrt(a*(1+b*txvec^2))
for (i in seq(l)) {
abveci<-abvec[i]
ncpi<-psis/abveci
lt<-qt(dd,df,ncpi)
ut<-qt(1-dd,df,ncpi)
intlt<-(ut-lt)/numint
tvec<-lt+intlt*(0:numint)
wtpdf<-(intlt/3)*coevec*dt(tvec,df,ncpi)
psisl<-rep(0,l)
psisu<-rep(0,l)
for (j in seq(l)) {
psisl[j]<-tnonct(tvec[j],df,alphau)*abveci
psisu[j]<-tnonct(tvec[j],df,alphal)*abveci}
wvec<-psisu-psisl
quan[i]<-sum(wtpdf*(wvec<omega))}
eap<-sum(wtxpdf*quan)}
else {
fvec<-bvec/(b*(1-bvec))
bpdf<-df(fvec,p,dfx,thetaa)/(b*(1-bvec)^2)
wbpdf<-(intbl/3)*coevec*bpdf
abvec<-sqrt(a*(1+b*fvec))
for (i in seq(l)) {
abveci<-abvec[i]
ncpi<-psis/abveci
lt<-qt(dd,df,ncpi)
ut<-qt(1-dd,df,ncpi)
intlt<-(ut-lt)/numint
tvec<-lt+intlt*(0:numint)
wtpdf<-(intlt/3)*coevec*dt(tvec,df,ncpi)
```

```
psisl<-rep(0,l)
psisu<-rep(0,l)
for (j in seq(l)) {
psisl[j]<-tnonct(tvec[j],df,alphau)*abveci
psisu[j]<-tnonct(tvec[j],df,alphal)*abveci}
wvec<-psisu-psisl
quan[i]<-sum(wtpdf*(wvec<omega))}
eap<-sum(wbpdf*quan)}}

n<-9
eap<-0
while(eap<ap & n<1001){
n<-n+1
nvec<-n*rvec
nt<-sum(nvec)
eap<-findtbap()}
print("group sizes, nt, attained assurance probability")
print(c(nvec,nt,eap),digits=6)}
===================================
> ancova.scie.apx3.fun(alpha=0.05, g=3, p=1, sigsq=1, rvec=c(1,1,1),
cvec=c(1,-0.5,-0.5), psis=0.5, theta=0, omega=1.5, ap=0.8)

[1] "g, p, alpha, psis, theta, omega, ap, rvec, cvec"
 [1]  3.00  1.00  0.05  0.50  0.00  1.50  0.80  1.00  1.00  1.00  1.00
-0.50 -0.50
[1] "group sizes, nt, attained assurance probability"
[1] 12.000000 12.000000 12.000000 36.000000  0.889952
```
